# Identification of tissue-enriched novel transcripts and novel exons in mice

**DOI:** 10.1186/1471-2164-15-592

**Published:** 2014-07-13

**Authors:** Seong-Eui Hong, Hong Ki Song, Do Han Kim

**Affiliations:** School of Life Sciences and Systems Biology Research Center, Gwangju Institute of Science and Technology (GIST), 123 Cheomdangwagi-ro (Oryong-dong), Buk-gu, Gwangju, 500-712 Korea

**Keywords:** Next generation sequencing, Novel transcript, Novel exon, Tissue-specificity

## Abstract

**Background:**

RNA sequencing (RNA-seq) has revolutionized the detection of transcriptomic signatures due to its high-throughput sequencing ability. Therefore, genomic annotations on different animal species have been rapidly updated using information from tissue-enriched novel transcripts and novel exons.

**Results:**

34 putative novel transcripts and 236 putative tissue-enriched exons were identified using RNA-Seq datasets representing six tissues available in mouse databases. RT-PCR results indicated that expression of 21 and 2 novel transcripts were enriched in testes and liver, respectively, while 31 of the 39 selected novel exons were detected in the testes or heart. The novel isoforms containing the identified novel exons exhibited more dominant expression than the known isoforms in heart and testes. We also identified an example of pathology-associated exclusion of heart-enriched novel exons such as *Sorbs1* and *Cluh* during pressure-overload cardiac hypertrophy.

**Conclusion:**

The present study depicted tissue-enriched novel transcripts, a tissue-specific isoform switch, and pathology-associated alternative splicing in a mouse model, suggesting tissue-specific genomic diversity and plasticity.

**Electronic supplementary material:**

The online version of this article (doi:10.1186/1471-2164-15-592) contains supplementary material, which is available to authorized users.

## Background

Information on the spatial and temporal signatures of transcriptomes is essential for diagnosis and treatment of severe diseases such as cardiomyopathies and malignant cancers. For the past several decades, high-throughput (HTP) data generated using the microarray method have contributed significantly to the discovery of quantitative signatures of various diseases. However, the microarray method has critical limitations, such as spatial bias, uneven probe problems, low sensitivity, and dependency on the probes spotted. Therefore, large-scale transcriptomic analyses using the microarray method have been superseded by the RNA-Seq generated through application of the recently developed next-generation sequencing (NGS) method.

RNA-Seq is a revolutionary method useful for transcriptomic signatures, since it can elucidate both quantitative and qualitative signatures (e.g., alternative splicing, AS) by *de novo* analysis, and it has therefore made possible the large-scale discovery of novel transcripts, such as noncoding RNAs. AS is an important event for proteome complexity and proteome diversity. However, current approaches using microarray or serial analysis of gene expression (SAGE) tags have faced limitations, such as probe dependency and low coverage. The robust sequencing capacity of RNA-Seq has dramatically increased our knowledge of dynamic alternation via AS. For instance, RNA-seq has revealed the subtype-specific novel isoforms for the most common breast cancers (e.g. triple negative breast cancer (TNBC), non-TNBC, and human epidermal growth factor receptor 2 (HER2)-positive breast cancer [1]). Information related to novel exons, recognized in the intronic regions, has rapidly increased owing to RNA-Seq [2–4]. *De novo* analyses of RNA-Seq datasets have rapidly updated the genome annotations of different species through examination of novel transcripts [5–7]. Furthermore, the detection of novel non-coding RNAs by RNA-Seq has identified them as important functional molecules regulating various biological processes [8–10].

The present study employed RNA-seq data to identify novel exons and novel transcripts enriched in different tissues in mice (here “novel” means “new” exons or “new” transcripts not identified in mice so far), leading to the discovery of novel transcripts expressed in testes or liver, and recognition that the novel isoforms containing the novel exons were dominantly expressed in testes or heart. These results should contribute to a more sophisticated annotation of the mouse genome, as well as improved understanding of tissue-specific gene regulation.

## Results and Discussion

### *In silico*analysis of tissue-enriched novel transcripts and exons

In order to identify tissue-enriched novel transcripts and exons in mice, the RNA-seq datasets for six tissues (i.e., GSE30352 for brain, cerebrum, heart, kidney, liver and testes) [11] were analyzed using the pipeline ‘Tophat-Cufflinks-Cuffcompare’ [12, 13]. As a result, 76,250 and 77,784 transcribed loci were constructed using UCSC and ENSEMBL, respectively. Among the transcribed loci, 184 transcripts located in the intergenic region were collected as putative novel transcripts (Additional file 1: Table S1). From this list of putative novel transcripts, we further examined the tissue-enriched transcripts using DESeq [14]. Novel transcripts exhibiting significant enrichment (*P* < 0.05) in the specific tissue were eventually defined as tissue-enriched novel transcripts. As a result, 32 and 2 novel transcripts were found to be significantly enriched in testes and liver, respectively (Table 1).

**Table 1:** **Summary of novel exons**

ID	Position (mm9)		# of exons	Tissue	***p***-value min ^1^	***p***-value max ^2^	Homologous protein	Non-mouse gene	EST
tNT-1	chr1:99242925-99375659	+	13	Testis	2.82E-10	2.38E-06	XP_001475034.3	Slc06d1	-
tNT-2	chr1:163105753-163167403	+	18	Testis	2.17E-07	0.000138	XP_344166.4	Slc9c2	-
tNT-3	chr1:121786157-121796422	-	5	Testis	0.000605	0.0083	NP_001102853	-	-
tNT-4	chr10:86118523-86133752	+	9	Testis	6.79E-07	6.28E-05	XP_487135.3	-	-
tNT-5	chr10:85157135-85173678	-	10	Testis	4.18E-05	0.002125	XP_896558.3	-	-
tNT-6	chr10:85989049-86004244	-	9	Testis	2.73E-07	9.99E-06	XP_896769.1	-	-
tNT-7	chr10:111578812-111597369	-	6	Testis	1.13E-06	0.000297	XP_001480681.1	-	-
tNT-8	chr13:56527964-56535585	+	5	Testis	1.49E-08	1.44E-05	XP_001475551	RGD1562024	O
tNT-9	chr13:97569388-97679749	+	17	Testis	3.30E-08	2.86E-06	XP_005065555	Ankrd31	O
tNT-10	chr15:25984096-25992242	-	5	Testis	0.000857	0.014133	-	-	O
tNT-11	chr15:76363652-76365439	-	5	Testis	1.13E-08	5.45E-05	XP_988010.2	Tmem249	O
tNT-12	chr18:13666918-13682257	+	5	Testis	0.000183	0.005416	YP_480919	-	-
tNT-13	chr18:32317886-32322406	-	5	Testis	4.44E-07	0.000236	WP_005016571	-	O
tNT-14	chr18:32617748-32622397	-	8	Testis	0.000202	0.014407	ELW62217	-	O
tNT-15	chr19:40823242-40903550	+	22	Testis	4.82E-08	0.001078	XP_004749675	-	O
tNT-16	chr2:170290671-170296220	-	5	Testis	4.46E-08	0.000182	XP_004246409	-	O
tNT-17	chr2:173112853-173116672	-	5	Testis	1.93E-06	0.00039	YP_003981506	-	O
tNT-18	chr3:31543868-31589424	-	14	Testis	1.07E-07	6.66E-05	NP_001028651.1	-	-
tNT-19	chr5:28278582-28303869	+	6	Testis	6.41E-11	1.92E-06	XP_003085546	-	-
tNT-20	chr5:129869449-129872910	+	5	Testis	9.84E-09	2.13E-05	YP_001641156	-	O
tNT-21	chr5:117435484-117468048	-	5	Testis	0.000816	0.017383	XP_001524870.1	-	O
tNT-22	chr6:16406558-16419928	-	6	Testis	7.12E-05	0.00307	-	-	O
tNT-23	chr6:44030493-44033356	-	5	Testis	0.001282	0.018015	-	-	O
tNT-24	chr7:120126477-120132935	+	5	Testis	0.004419	0.041732	EGV91268	-	O
tNT-25	chr7:127696533-127711472	+	5	Testis	7.17E-05	0.002835	EDL17209.1	-	O
tNT-26	chr7:36029889-36060558	-	10	Testis	1.14E-08	4.01E-06	XP_001480194	WDR88	-
tNT-27	chr8:74348600-74377254	-	11	Testis	9.06E-06	0.001105	EDL28738	-	O
tNT-28	chrX:98891146-98901100	+	5	Testis	0.00037	0.034496	XP_005095122	-	-
tNT-29	chrX:43597928-43606686	-	5	Testis	1.97E-10	1.94E-06	-	-	-
tNT-30	chr12:44067864-44135252	-	5	Testis	0.002254	0.017821	EDL38698.1	-	
tNT-31	chr17:14128560-14192168	-	6	Testis	6.01E-11	4.13E-06	EDL20486.1	-	-
tNT-32	chr17:21191727-21199635	-	6	Testis	7.30E-11	3.14E-06	EDL20488.1	-	O
lNT-1	chr10:111026064-111048582	+	5	Liver	1.19E-05	0.021035	EDL21734.1	-	O
lNT-2	chr12:73709901-73729881	-	6	Liver	4.69E-06	0.009759	XP_003512062.1	Dhrc7	O

^1,2^Indicate the minimum and maximum *p*-values, respectively, when the expression of novel transcripts in testis compared to other tissues in a pairwise manner.

In addition to the novel transcripts, we examined the tissue-enriched novel exons for known genes and the novel junctions for the obtained *de novo* transcripts. In total, 5,582 novel exons were identified from 6 tissues (Additional file 2: Table S2). To examine tissue-enrichment of the novel exons, the read numbers for the novel exons were counted and compared across the 6 tissues in a pairwise manner using DESeq. Of the 236 novel exons evaluated, 197 were expressed in testes (Additional file 2: Table S2), which was consistent with a study by Howald *et al.* reporting that these novel transcripts are mainly identified in the testes of humans [15].

### Experimental confirmation of testes- and liver-enriched novel transcripts

**Figure 1:**
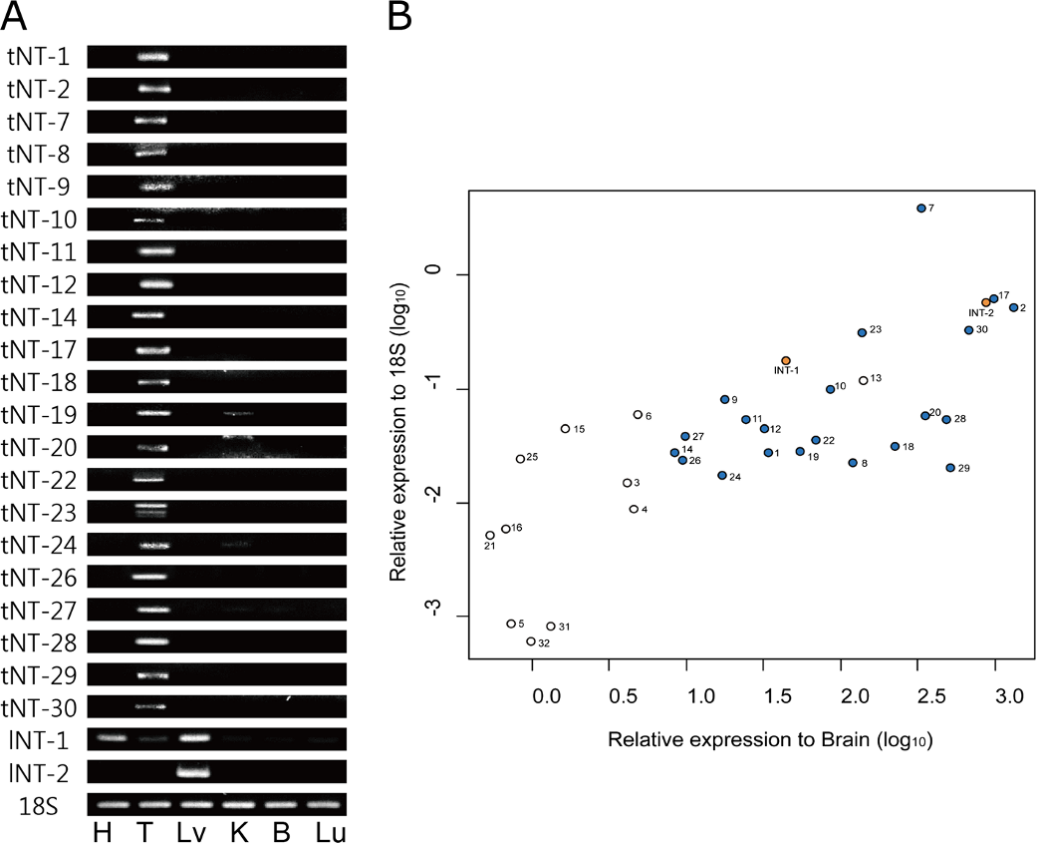
**Testes- and liver-enriched expression of the novel transcripts.** The expressions of testes- and liver-enriched novel transcripts were experimentally confirmed by **(A)** RT-PCR and **(B)** qRT-PCR for 6 tissues (H: Heart, T: Testes, Lv: Liver, K: Kidney, B: Brain, Lu: Lung). tNT and lNT indicate testes- and liver-enriched novel transcripts, respectively. tNTs and lNTs with blue and orange circles, respectively, were experimentally confirmed by both RT-PCR and qRT-PCR experiments. Values on the X axis indicate the relative expression of tNTs and lNTs in testes and liver, respectively, compared to the expressions in brain (log10(2-△△Ct)). The values on the Y axis indicate the relative expression of the novel transcripts when compared to the expressions of 18S in testes (log10(2-△Ct)).

Enrichment of the putative novel transcripts in testes and liver was further examined experimentally using mouse heart, testes, liver, kidney, brain and lung tissues by qRT-PCR and RT-PCR, to determine the expression levels and patterns. Among the 32 testes- and 2 liver-enriched novel transcripts (32 tNT and 2 lNT), enrichment of 21 tNTs and 2 lNTs were experimentally confirmed (Figure 1). We were unable to detect 11 tNTs, including tNT-5, −31, and −32, by RT-PCR. Although highly specific expression of tNT-13 was found in testes using qRT-PCR, we could not detect the expression using RT-PCR, which may have been due to low expression levels.

Expression levels of tNTs and lNTs were generally enriched in testes and liver (e.g. 8.3–1,328-fold higher than in the brain). However, the most specific expression in testes was observed in tNT-2 (1,328–3,502 fold higher than other tissues), a homolog to *Slc9c2*, which is a human Na^+^/H^+^ exchanger. tNT-7 was the most abundantly expressed of the tNTs (Figure 1B). No expressed sequence tag (EST) for tNT-7 has been reported to date, however, it is predicted to be homologous to cysteine-rich secretory protein (CRISP) involved in sperm-egg fusion [16]. Most of the tNTs encoding proteins with MW values ranging from 6–389 kDa exhibited a broad range of similarity (19–100%) between the species (Table 1). Despite the absence of a matched mouse gene or EST, tNT-1 was identical to the predicted protein model, XP_001475034.3, and shared high sequence identity with rat *Slco6d1* (~80%), suggesting that it may function as an ion transporter in testes. tNT-18 seems to encode a protein identical to NP001028651.1 encoded by *Gm1516* in chromosome 3. tNT-18 is located 3Mbps away from *Gm1516* in chromosome 3, indicating that *Gm1516* and tNT-18 are paralogs encoding the same protein sequence.

Many of these novel transcripts are predicted to encode functional domains or highly homologous proteins in other species, as well (Table 1). Conversely, two testes-enriched novel transcripts (tNT-10 and −22) likely represented noncoding transcripts. Noncoding transcripts are also important regulatory molecules involved in diverse processes such as gene-specific transcription [17], regulation of basal transcriptional machinery [18], splicing [19], and translation [20]. The in-depth functional characterization of the confirmed testes- and liver-enriched novel transcripts is expected to lead to important information regarding tissue-specific gene regulation.

### Experimental confirmation of testes-enriched novel exons

**Figure 2:**
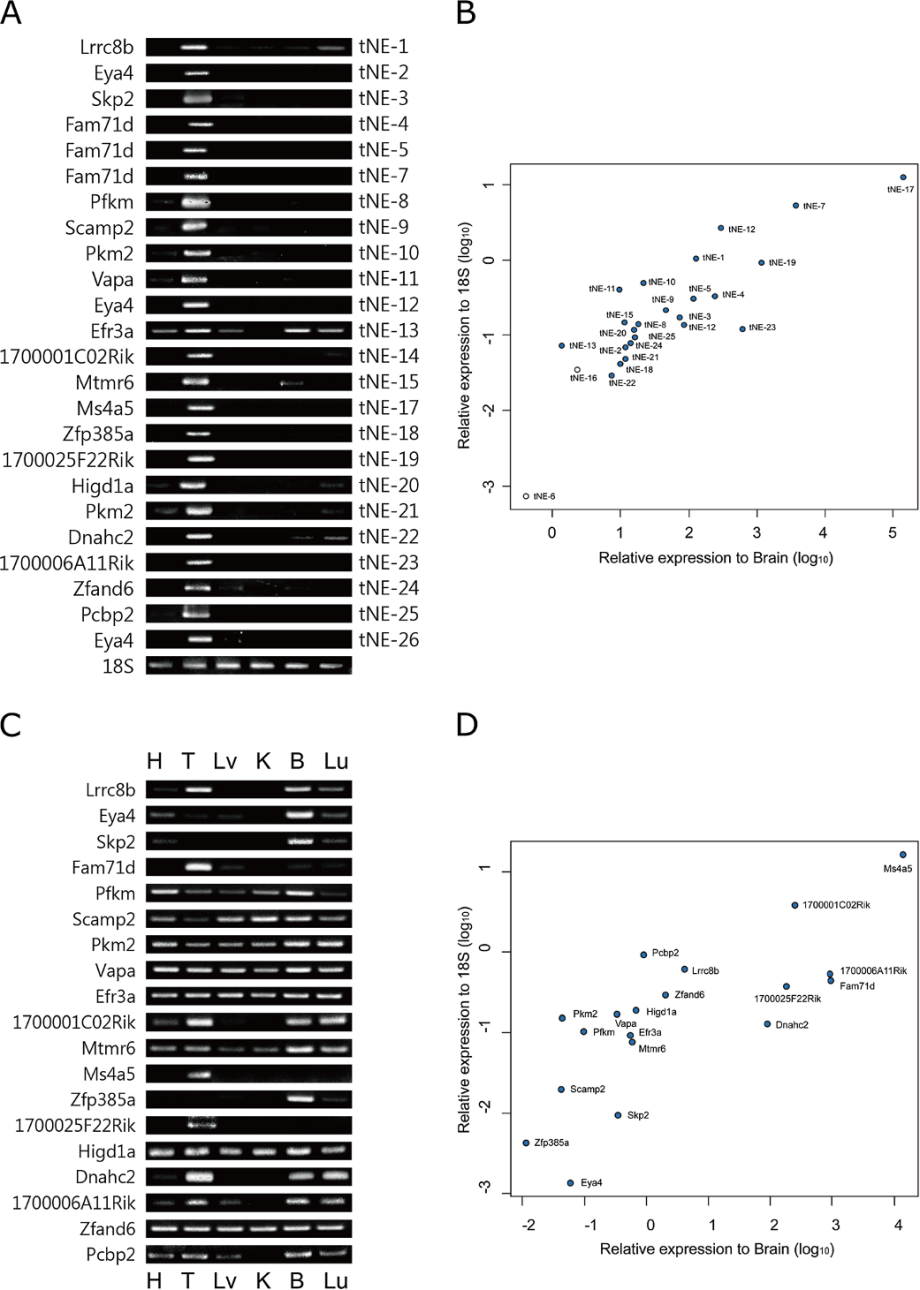
**Testes-enriched novel exons.** The expressions of testes-enriched novel exons (tNEs) were experimentally confirmed by **(A)** RT-PCR and **(B)** qRT-PCR. Blue circles indicate the tNEs confirmed by RT-PCR. The expression levels of the genes containing tNEs were measured by **(C)** RT-PCR and **(D)** qRT-PCR.

Among 197 testis-enriched novel exons, 26 novel exons were selected for experimental validation, on the basis of their read number (expression level), easiness of primer design, and straightforward exon structures. Among the 26 testes-enriched novel exons (hereafter, tNE), the strong enrichment of 24 tNEs in testes was confirmed by qRT-PCR and RT-PCR (Figure 2A and B). tNE-17 of *Ms4a5* was the most abundantly and specifically expressed in testes, whereas tNE-6 was barely expressed in testis. tNE-1, −13, −15 and −22 were strongly expressed in testes, whereas little or no expression was observed in other tissues. Multiple novel exons were identified for *Eya4* (tNE-2, −12 and −26), *Fam71d* (tNE-4, −5 and −7) and *Pkm2* (tNE-10 and −21). We further examined the expression of the genes containing tNEs to determine whether the expression was due to testes-specific genes. Results indicated that most of the genes containing tNEs were ubiquitously expressed in different tissues (Figure 2C and D). However, the expressions of genes such as *Skp2*, *Eya4*, *Scamp2*, and *Zfp385a* were significantly lower in testes than in the brain (Figure 2D), despite strong expression of the tNEs (i.e., tNE-2, −3, −9, −12, −18 and −26) in testes, while the strong expressions of tNEs of *Fam71d*, *Ms4a5* and *1700025F22Rik* were assumed to be due to the testis-specific expression of the genes.

We hypothesized that the insertion of novel exons could produce new UTRs or protein variants, as listed in Table 2. More than half of the testes-enriched novel exons (n = 112, 56.8%) were identified as alternative 5′-UTRs that would likely result in the differential regulation of transcription or translation in testes. Several studies have demonstrated that testes-specific 5′-UTRs include regulatory elements, such as the upstream open reading frames (uORFs), for translational regulation [21–23]. We also found that testes-enriched novel 5′-UTRs have abundant uORFs (n = 56, 50%) with some of 197 novel exons in the testes, suggesting a testes-specific regulatory role in translation. For example, more than 5 uORFs were found to be the testis-enriched 5′-UTRs of *Nt5c2*, *Lrrc8b*, *Mllt11*, *Mphosph9*, *Kdm5b*, *Proca1*, and *5730559C18Rik* in 197 testis-enriched novel exons. Additionally, the inclusion of tNE-2, 3, 20 and 21 of *Eya4*, *Skp2, Higd1a* and *Pkm2* could contribute to the 5′UTRs forming G-quadruplex, which is involved in translational control [24].

**Table 2:** **Summary of testis or heart-enriched novel exons**

ID	Gene	Position (mm9)	Length		Protein	Prediction ^1^
hNE-1	Cluh	chr11:74467029-74467303	275	+	Q5SW19	38 AA shorter
hNE-2	Mylk4	chr13:32820204-32820624	421	-	Q5SUV5	
hNE-3	Clasp1	chr1:120451862-120451963	102	+		
hNE-4	Schip1	chr3:68388089-68388346	258	+	Q3TI53	27 AA different
hNE-5	Mylk4	chr13:32868030-32868501	472	+	Q5SUV5	85 AA different
hNE-6	Mylk4	chr13:32818712-32819001	290	+	Q5SUV5	
hNE-7	Clasp1	chr1:120378050-120378669	620	+		
hNE-8	Trdn	chr10:33086092-33087214	1123	+		Same as 51 kDa skeletal Trdn
hNE-9	Sorbs1	chr19:40452144-40452869	726	-	Q62417	241 AA longer
hNE-10	Csde1	chr3:102840498-102840644	147	+	Q91W50	46 AA longer
hNE-11	Larp5	chr13:9127241-9130370	3130	+	Q80UQ3	105 AA longer
hNE-12	Nedd5l	chr18:65243095-65244448	1354	+		
hNE-13	Nexn	chr3:151927873-151928180	308	-	Q7TPW1	
tNE-1	Lrrc8b	chr5:105881814-105883128	1315	+	Q5DU41	Different 5′UTR
tNE-2	Eya4	chr10:22905057-22905389	333	-	Q9Z191	Different 5′UTR
tNE-3	Skp2	chr15:9082539-9082780	242	-	Q9Z0Z3	Different 5′UTR
tNE-4	Fam71d	chr12:79824797-79824939	143	+	D3YV92	26 shorter AA, different C term
tNE-5	Fam71d	chr12:79796826-79797030	205	+	D3YV92	Different 5′UTR
tNE-6	Rfx1	chr8:86608465-86608794	330	+		
tNE-7	Fam71d	chr12:79823117-79823280	164	+	D3YV92	
tNE-8	Pfkm	chr15:97925522-97925641	120	+	Q1LZL7	70 A.A longer
tNE-9	Scamp2	chr9:57426081-57426209	129	+	Q9ERN0	44 AA longer
tNE-10	Pkm2	chr9:59510847-59510963	117	+	P52480	Different 5′UTR
tNE-11	Vapa	chr17:65936384-65936506	123	-	Q9WV55	41 AA longer
tNE-12	Eya4	chr10:22903219-22903421	203	-	Q9Z191	Different 5′UTR
tNE-13	Efr3a	chr15:65696232-65696453	222	+	Q8BG67	131 AA shorter (C-term)
tNE-14	1700001C02Rik	chr5:30779031-30779154	124	+	Q9DAS2	N-term 15 AA
tNE-15	Mtmr6	chr14:60909543-60909656	114	+	Q8VE11	38 AA longer
tNE-16	Mbtd1	chr11:93800835-93801026	192	+		
tNE-17	Ms4a5	chr19:11352451-11352587	137	-	Q810P8	C-term 97 AA shorter
tNE-18	Zfp385a	chr15:103151501-103151619	119	-	Q8VD12	N-term 50 AA shorter
tNE-19	1700025F22Rik	chr19:11233536-11233685	150	-	Q6P8I0	56 AA longer
tNE-20	Higd1a	chr9:121765839-121765990	152	-	Q9JLR9	Different 5′UTR
tNE-21	Pkm2	chr9:59506806-59506960	155	+	P52480	Different 5′UTR
tNE-22	Dnahc2	chr11:69331069-69331230	162	-	Q9P225	54 AA longer
tNE-23	1700006A11Rik	chr3:124105142-124105398	257	-	B9EHI3	71 AA longer (C-term)
tNE-24	Zfand6	chr7:91790796-91790947	152	-	Q9DCH6	Different 5′UTR
tNE-25	Pcbp2	chr15:102303428-102303532	105	+	Q61990	Different 5′UTR
tNE-26	Eya4	chr10:22902544-22902630	87	-	Q9Z191	31 AA shorter

^1^Changes of amino acid sequences and UTRs due to insertions of novel exons were predicted using the free software ‘Translate’ provided by ExPASy [39].

Insertions of tNEs may lead to dramatic changes in protein expression. (Example 1) The C-terminal truncation (~50%) of MS4A5 is related to the insertion of tNE-17. MS4A5 is known to have four membrane-spanning domains [25], but insertion of tNE-17 results in the loss of two domains. (Example 2) Prediction by cNLS mapper [26] suggests that the novel isoform of EFR3A lacks the C-terminal 131 residue sequence containing one of the nuclear localization signals (NLSs). It is also possible that tNE-13 plays an important role in the regulation of EFR3A localization in testes [27]. (Example 3) For tNE-11 belonging to *Vapa*, two variants showing a 9-bp difference were identified by Cufflinks (Additional file 3: Figure S1A) and were predicted to encode 38–41 additional amino acids, GKTPPGIASTVASLSSVSSAVATPASYHLKNDPRELKE (VKQ). Interestingly, it is likely that this sequence contributes to the membrane-spanning region in a testes-specific manner by the prediction using TopPred [27] (Additional file 3: Figure S1B). The function of VAPA in neurons is known to be associated with ER and microtubules [28], and tNE-11 might confer testes-specific functions via the membrane-spanning region. Collectively, these data suggest that the testes-enriched novel exons could be involved in dramatic structural changes.

### Experimental confirmation of the heart-specific novel exons

**Figure 3:**
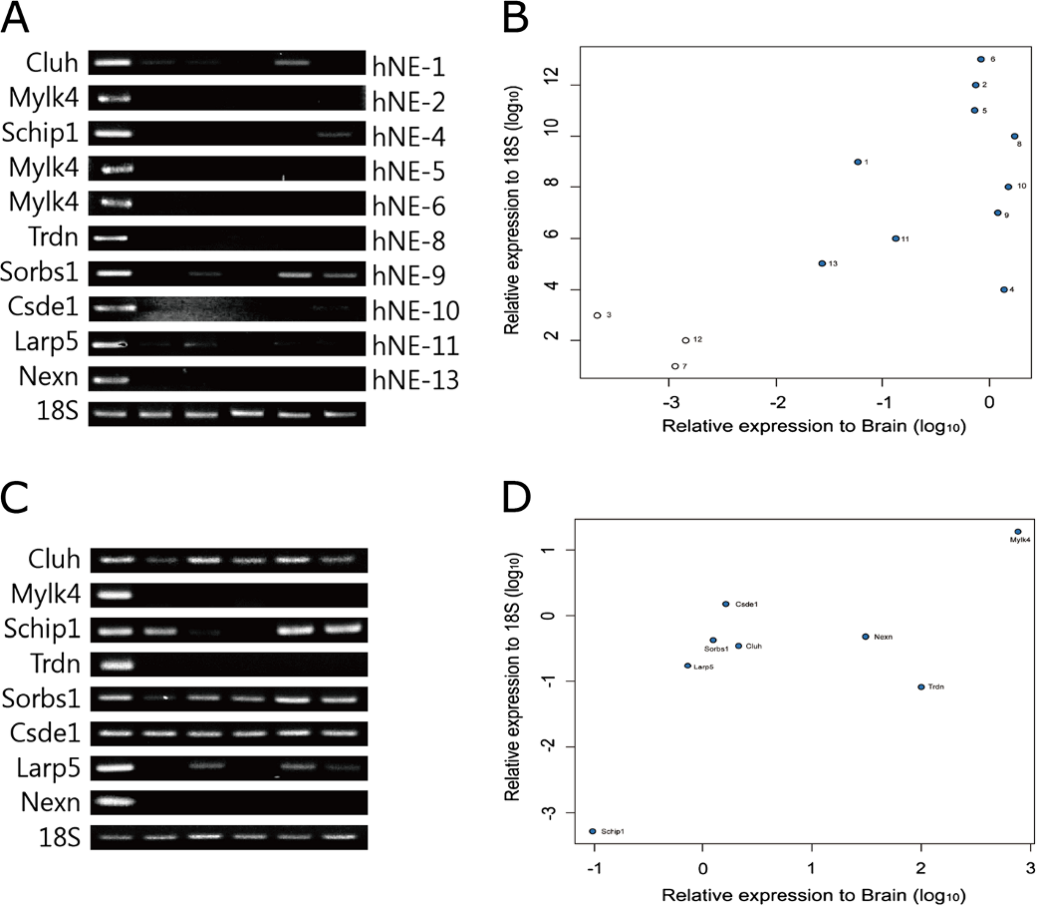
**Heart-enriched novel exons.** The expressions of heart-enriched novel exons (hNEs) were experimentally confirmed by **(A)** RT-PCR and **(B)** qRT-PCR. The expression levels of the genes containing the hNEs were measured by **(C)** RT-PCR and **(D)** qRT-PCR.

Among 26 heart-enriched novel exons, 13 novel exons (hereafter, hNE) were selected for experimental validation, on the basis of their read number (expression level), easiness of primer design, and straightforward exon structures and the enrichment of 10 hNEs in heart was experimentally confirmed by qRT-PCR and RT-PCR (Figure 3C and D). Most hNEs were strongly expressed in the heart, except for hNE-1 and −9. Multiple novel exons (i.e., hNE-2, −5 and −6) were identified in *Mylk4* and predicted to produce a different 5′UTR with slightly different N-terminal regions. Similar to the tNEs, the alternative 5′-UTRs containing 1–2 uORFs were observed in the hNEs for *Cluh*, *Mylk4*, *Schip1*, *Larp5*, and *Nexn*, suggesting heart-specific post-transcriptional regulation.

Among the variants identified, hNE-8 of *Trdn* is likely to result in truncation of the C-terminal region. A total of six isoforms were identified for *Trdn*, and their estimated sizes were approximately 1.3, 4.3, and 5 kb in the heart, and 5, 5.5, and 7 kb in skeletal muscle [29]. In addition, hNE-8 was specifically expressed in the heart and inserted in the transcripts expressed in skeletal muscle, which could result in the C-terminus-truncated TRDN. Based on analysis of data using Cufflinks, the relative expression of the isoform containing hNE-8 was predicted to be considerably lower than the known cardiac-specific isoforms (Additional file 4), suggesting a restricted role for hNE-8 of *Trdn* in the heart.

Several dramatic changes were predicted in the case of *Sorbs1* variants containing hNE-9. This predicted additional exon was highly enriched in proline residues such as *PPP*A*PPP*D*PP*, *PP*CL*P*F*P*, *P*K*P*YI*PP*ST*P*, and *P*SL*P*T*P*TSV*P*. Proline-rich residues was known to be important for binding the SH3 domains in signaling cascades [30, 31], therefore it suggested the insertion of hNE-9 might be involved in the regulation of signaling cascade in a heart-specific manner. At present, seven known isoforms of *Sorbs1* have been identified [32–34] and hNE-9 is novel and appears highly enriched in the heart. Additionally, data suggested that hNE-10 from *Csde1* likely encoded a serine-rich region consisting of 46 additional residues (MENMLTV*SS*DPQPTPAAPP*S*L*S*LPL*SSSS*T*SS*WTKKQKRTPTYQR*S)*. Interestingly, Ser-32 and Thr-34 of hNE-10 were predicted to be phosphorylated by PKC according to NetPhosK [35], suggesting heart-specific signal regulation.

### Alternative splicing patterns of the novel isoforms containing the novel exons

**Figure 4:**
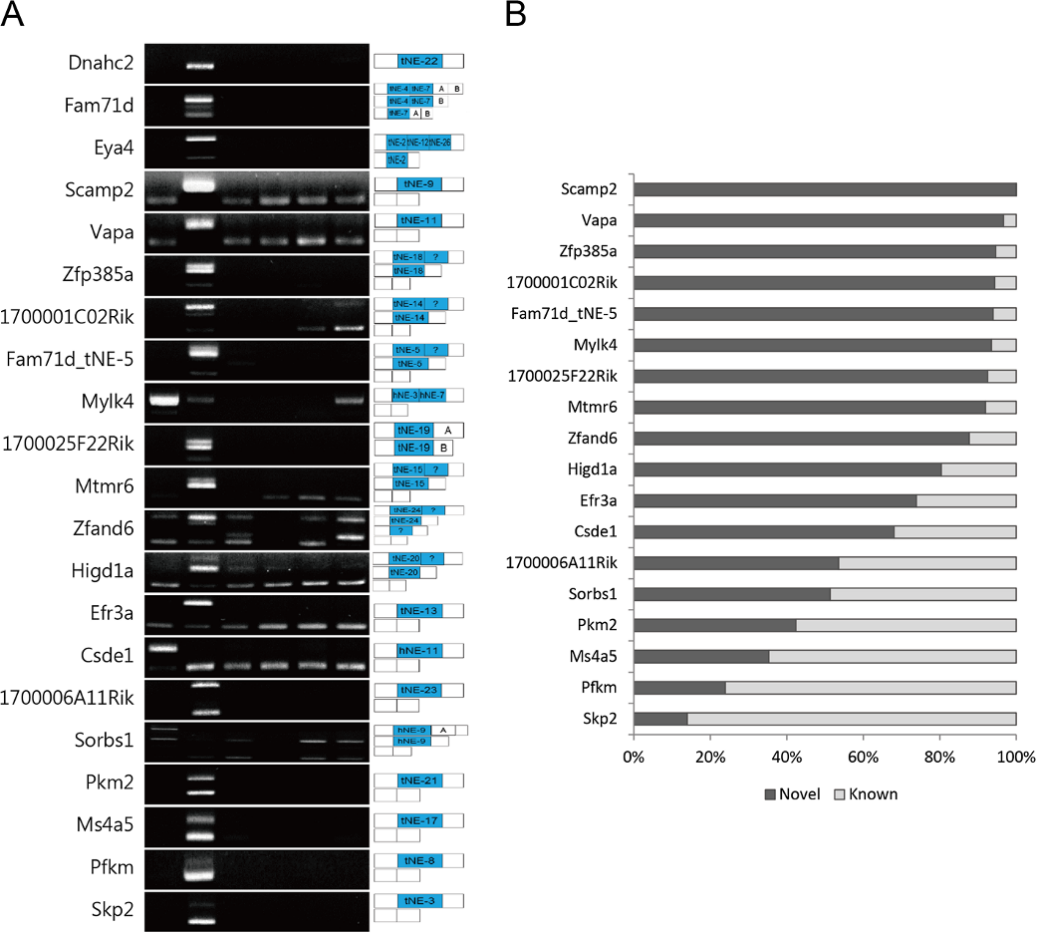
**Alternative splicing patterns of the novel isoforms containing the novel exons. (A)** Multiple isoforms either with or without the novel exons were produced by RT-PCR. Exon structures amplified by the primers are seen in the right panel, while novel exons are highlighted with blue boxes. **(B)** The relative expression levels of the isoforms were quantified by ImageJ.

We then compared the expression levels of the novel isoforms containing the novel exons to those of the known isoforms. As seen in Figure 4, at least 10 novel isoforms exhibited dominant expression when compared with the previously known isoforms in the heart or testes. More than 90% of the expressions of *Scamp2*, *Vapa*, *Zfp385a*, *1700001C02Rik*, *Fam71d*, *1700025F22Rik*, and *Mtmr6* were identified in the novel isoforms in testes, suggesting testes-specific roles of the isoforms. For *Mtmr6*, a recent study reported that the testes-specific MTMR6 protein had a slightly higher molecular weight than the known protein, but the similar portions of the novel and known isoforms were observed at a protein level [36].

Conversely, the novel isoforms for *Pkm2*, *Ms4a5*, *Pfkm*, and *Skp2* were expressed at relatively low levels in the testes. Unexpected isoforms were observed in *Zfp385a*, *1700001C02Rik*, *Fam71d, Mtmr6*, *and Zfand6*, implying incomplete coverage in spite of the high-resolution of NGS. However, the rapidly accumulating datasets will help complete a mouse gene annotation.

### Expressional changes of heart-specific novel exons during cardiac hypertrophy

**Figure 5:**
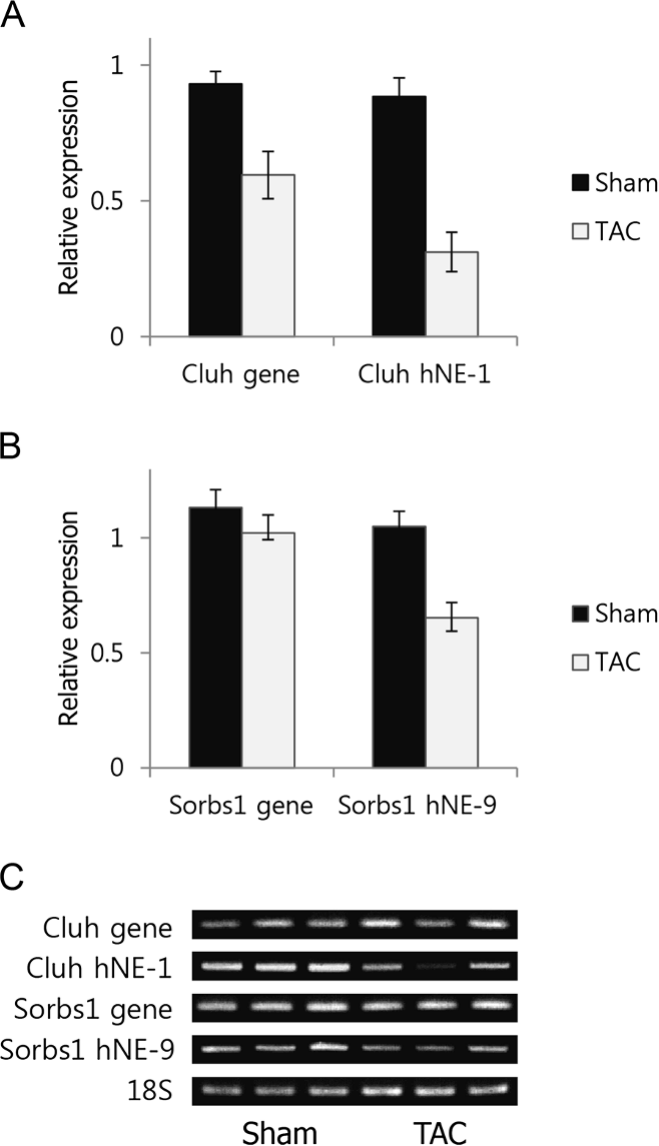
**Alternative splicing patterns of the novel exons of** ***Cluh*** **and** ***Sorbs1*** **during cardiac hypertrophy. (A)** The expression levels of *Cluh* and hNE-1, **(B)** *Sorbs1* and hNE-9, measured by qRT-PCR. Black and gray bars indicate the expression levels in Sham and TAC (n = 3 for each model), respectively, and **(C)** expression patterns measured by RT-PCR.

For the identified hNEs, we investigated the alternative splicing patterns occurring during cardiac hypertrophy induced by transverse aortic constriction (TAC). The number of reads mapped to all exons, including hNEs, were calculated using our RNA-Seq dataset (E-MTAB-727) on cardiac hypertrophy [37], and the differential expression levels of hNEs were identified using DEXSeq [38]. Two differentially expressed hNEs (hNE-1 and −9 for *Cluh* and *Sorbs1*, respectively) were obtained (*p* < 0.05) (Additional file 5: Table S3) from the analysis. As seen in Figure 5A, the expression of *Cluh* was significantly decreased by ~36% (*p* = 0.015), while the expression of hNE-1 decreased by ~65% during cardiac hypertrophy (*p* = 0.007), indicating that hNE-1 in *Cluh* was alternatively spliced during cardiac hypertrophy (gene vs. hNE-1 in TAC, *p* = 0.046). Cufflinks analysis (Additional file 6: Figure S3) indicated that the portion of the novel isoform containing hNE-1 represented approximately 33% of the expression of *Cluh* in the heart, and that the predicted protein derived from the isoform was 38 residues shorter than the known isoform. The expression of the heart-specific minor isoform containing hNE-1 was thought to be down-regulated during cardiac hypertrophy.

The expression of hNE-9 in *Sorbs1* was also significantly decreased during cardiac hypertrophy. While the expression of *Sorbs1* gene was not changed (*p* = 0.34), the expression of hNE-9 was significantly decreased by ~36% (*p* = 0.037) (Figure 5B). Thus, hNE-9 was thought to be excluded during cardiac hypertrophy suggesting a disease-related function associated with hNE-9 in the heart. Therefore, we examined the relationship between cardiac hypertrophy and hNEs, and further experimentally validated the significant exclusion of hNE-1 and −9 of *Cluh* and *Sorbs1* during TAC-induced cardiac hypertrophy. As no changes were indicated in exercise-induced cardiac hypertrophy (Table S3), we concluded that the exclusion of these exons could be related to pathology of the heart.

## Conclusions

The results of this study will contribute to updating mouse gene annotation through the identification of specific tissue-enriched novel transcripts and novel exons. Tissue-specific isoform switches mediated by novel exons could provide important insights into the tissue-specific roles of the novel exons. The exclusion of the hNEs during cardiac hypertrophy also suggested sensitivity of the novel exons to pathological status. Our findings emphasize the necessity of this approach to identify tissue-specific novel transcripts and exons.

## Methods

### Ethics Statement

All animal experiments and animal ethics were approved by the GIST Institutional Animal Care and Use Committee (IACUC) (Permit number: GIST-2013-22).

### Identification of novel transcripts and exons

‘Tophat-Cufflinks-Cuffompare’ pipeline was used to identify novel transcripts and exons. Fastq files for six mouse tissues (brain, cerebrum, kidney, heart, liver and testis) in GSE30352 were downloaded and the reads were further aligned to mouse genome (UCSC mm9 version) using ‘Tophat’. Using resultant Bam files, *de novo* assembly was performed to construct the transcripts using ‘Cufflinks’. All transcripts were then compared to the predefined gene annotations such as UCSC and ENSEMBL using ‘Cuffcompare’. To identify the novel transcripts, we collected the transcripts located in intergenic region and classified as “unknown” in both UCSC and ENSEMBL as putative novel transcripts. In case of novel exons, we searched the consecutive novel junctions in-between the known neighbouring exons, thereby deduced the novel exons spanning the novel junctions.

### Tissue-specificity of novel transcripts and exons

Numbers of the reads for the novel transcripts and exons were counted for heart, testis, liver, kidney, cerebrum and brain using HTSeq [39]. Multiple tests for the novel transcripts and exons were performed for a specific tissue vs. remaining tissues using DESeq and DEXSeq [14, 38], respectively, in a pairwise manner. The novel transcripts and exons significantly enriched in a specific tissue compared to all other tissues (*p* < 0.05) were collected.

### Alternative splicing of heart-enriched novel exons during cardiac hypertrophy

Differential expression of the heart-enriched novel exons during cardiac hypertrophy were analysed using ‘Tophat-HTSeq-DEXSeq’ pipeline. Fastq files of previously reported RNA-Seq datasets on TAC-induced cardiac hypertrophy were aligned to mouse genome (UCSC mm9 version) using Tophat [12]. Number of the reads mapped onto the genes containing the heart-enriched novel exons were counted using HTSeq. Then, the differential expression of the heart-enriched exons during cardiac hypertrophy were analysed using DEXSeq (*p* < 0.05).

### Transverse aortic constriction operation

Cardiac hypertrophy was induced by TAC operation under anesthesia with intraperitoneal injection of avertin, 2-2-2 tribromoethanol (Sigma, St. Louis, MO) dissolved in tert-amyl alcohol (Sigma, St. Louis, MO). The procedure of operation was followed as previously described [37]. As a control group, sham operation (same procedure except for tying) was done. 1 week after operation, mice were sacrificed, and hearts were removed, and then stored in deep freezer at -80°C before RNA extraction.

### Tissue preparation and RNA isolation

Adult (8 weeks old) C57BL6 mouse heart, testes, liver, kidney, brain and lung were snap frozen in liquid nitrogen, stores at -80°C, and homogenized in liquid nitrogen using a mortar and pestle. Approximately 450–700 mg of grinded whole mouse heart was used for extraction of total RNA with 1 ml Trizol Reagent® (Invitrogen, Carlsbad, CA) following the manufacturer’s instructions.

### RT-PCR and qRT-PCR

First-strand cDNA was synthesized from 2 μg of total RNA with Random hexamer using Omniscript® reverse transcription (Qiagen, Valencia, CA) according to the manufacturer’s instruction. Briefly, qRT-PCR assays were performed using TOPreal™ qPCR premix (Enzynomics, Korea) under the following two-step conditions: denaturation at 95°C for 15 seconds followed by annealing and extension at 60°C for 40 seconds, for a total of 40 cycles. The 18S transcript was used as an endogenous reference to assess the relative level of mRNA transcript. RT-PCR assays were performed on a ABI thermal cycler TP600 (TaKaRa, Japan) using nTaq-HOT DNA polymerase (Enzynomics, Daejeon, South Korea) under the following 3 step conditions: denaturation at 94°C for 30s, annealing at 55-60°C for 30s and extension at 72°C for 40s with total 35–37 cycles. All primer pairs are listed in Additional file 7: Table S4.

### Availability

GSE30352 and E-MTAB-727 are publicly available in the Gene Expression Omnibus (GEO) and European Nucleotide Archive (ENA) databases, respectively.

## Electronic supplementary material

Additional file 1: Table S1: Tissue-specific novel transcripts. Detailed information on 184 novel transcripts are listed. The novel transcripts were identified by the pipeline of ‘Tophat-Cufflinks-Cuffcompare’. Multiple isoforms transcribed from the same transcribed loci are in the list. (XLSX 30 KB)

Additional file 2: Table S2: List for the number of novel exons for the 6 tissues. (XLSX 10 KB)

Additional file 3: Figure S1: Structure of *Vapa* (A) Structures of *Vapa* and magnified image for the isoforms were illustrated using UCSC Genome browser (B) Predicted hydrophobicity of the novel exon of *Vapa* suggest the membrane spanning ability. TopPred was applied to predict hydrophobicity. (PNG 209 KB)

Additional file 4: Figure S2: Expression level of hNE-9 estimated by Cufflinks. (PNG 32 KB)

Additional file 5: Table S3: Expression levels of heart-specific novel exons (hNEs) during cardiac hypertrophy. Alternative splicing of hNEs during either transverse aortic constriction (TAC) or exercise-induced cardiac hypertrophy was determined using DEXSeq. (XLSX 10 KB)

Additional file 6: Figure S3: Relative expression levels of the isoforms for *Cluh* and *Sorbs1* in heart. Relative expression levels of the isoforms were measured by FPKM of Cufflinks. Green bars indicate the expression levels of the isoforms containing novel exons. (PNG 32 KB)

Additional file 7: Table S4: Primers used. (XLSX 21 KB)

## Abbreviations

NGS: Next generation sequencing
AS: Alternative splicing
SAGE: Serial analysis of gene expression
RNA-Seq: RNA Sequencing
TNBC: Triple negative breast cancer
HER: human epidermal growth factor receptor
UCSC: University of California, Santa Cruz
FPKM: Fragments per kilobase of exon per million fragments mapped
NLS: Nuclear localization signal
TAC: Transverse aortic constriction.
