# Supplementary figures and images for: Identification of tissue-enriched novel transcripts and novel exons in mice
Source: BMC Genomics. 2014 Jul 13;15(1):592. doi: 10.1186/1471-2164-15-592 (PMC4111849; doi:10.1186/1471-2164-15-592)

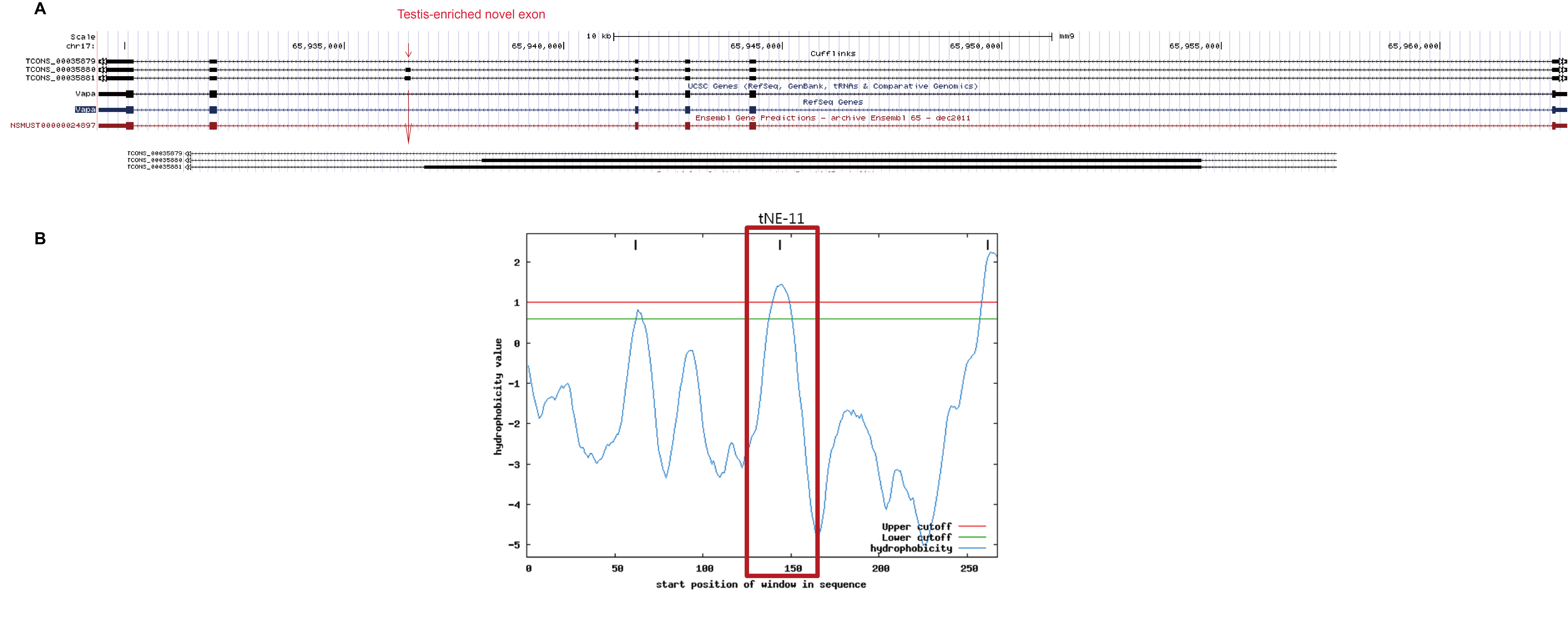

Supplement: Supplementary file 3 — Additional file 3: Figure S1: Structure of Vapa (A) Structures of Vapa and magnified image for the isoforms were illustrated using UCSC Genome browser (B) Predicted hydrophobicity of the novel exon of Vapa suggest the membrane spanning ability. TopPred was applied to predict hydrophobicity. (PNG 209 KB) [file 12864_2014_6277_MOESM3_ESM.png]

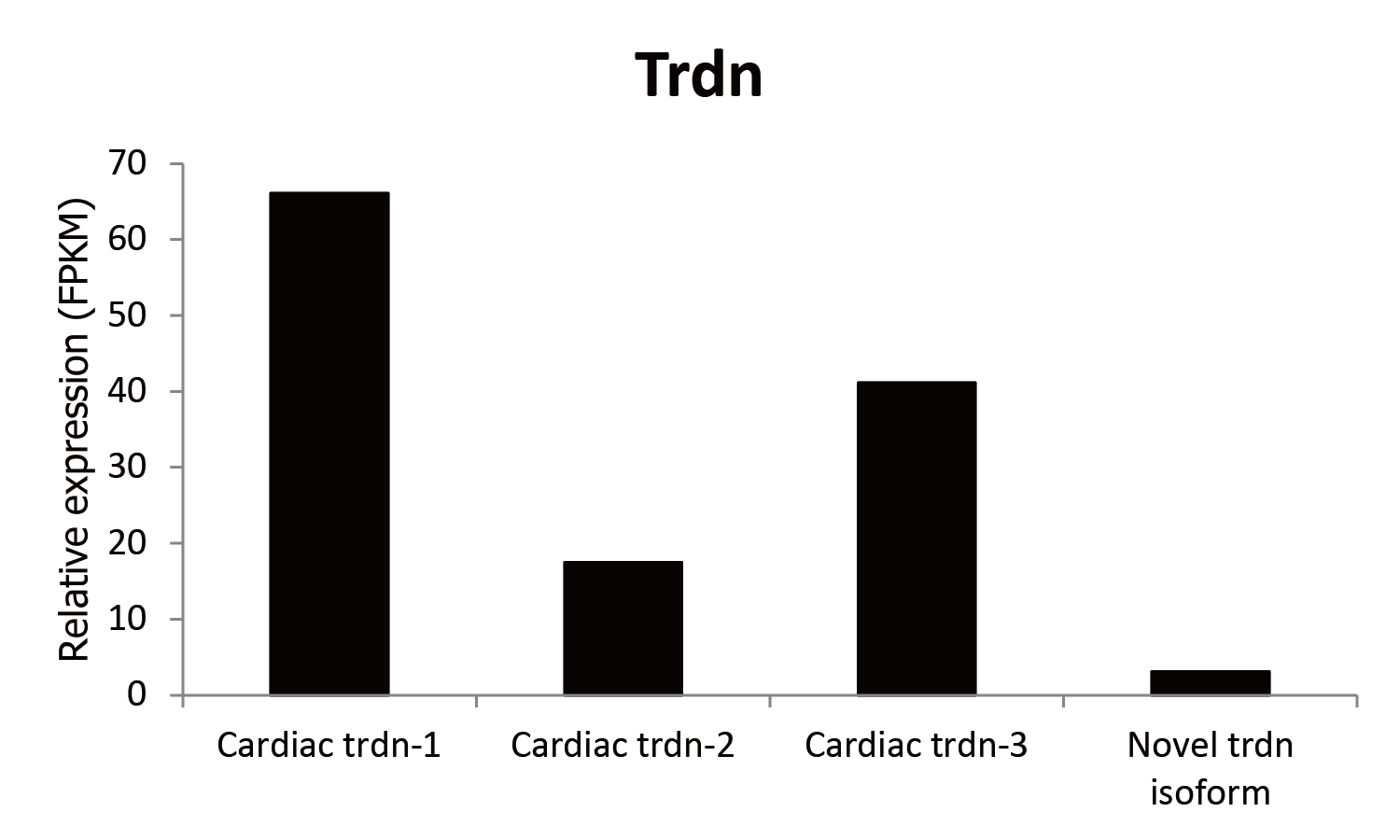

Supplement: Supplementary file 4 — Additional file 4: Figure S2: Expression level of hNE-9 estimated by Cufflinks. (PNG 32 KB) [file 12864_2014_6277_MOESM4_ESM.png]

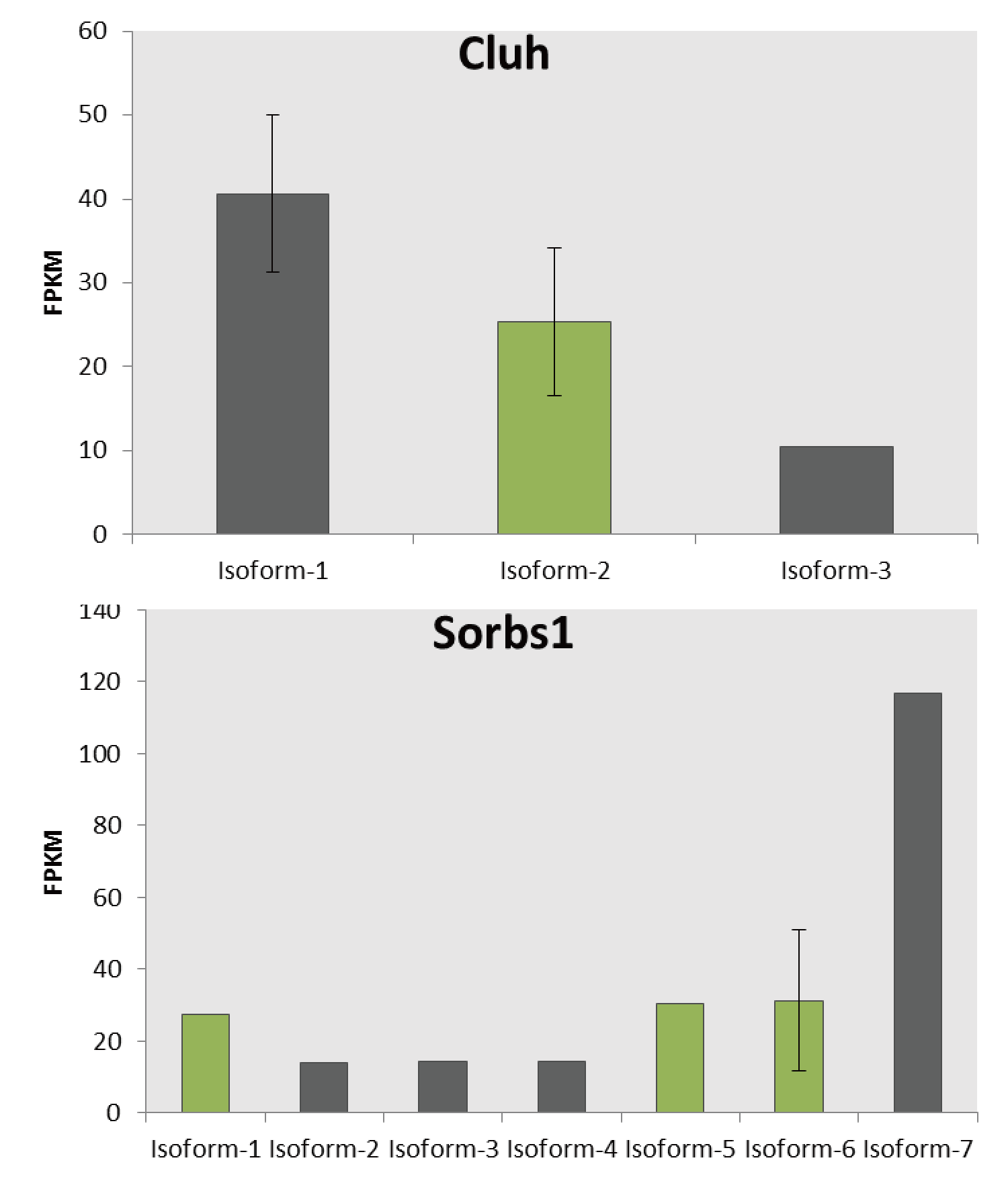

Supplement: Supplementary file 6 — Additional file 6: Figure S3: Relative expression levels of the isoforms for Cluh and Sorbs1 in heart. Relative expression levels of the isoforms were measured by FPKM of Cufflinks. Green bars indicate the expression levels of the isoforms containing novel exons. (PNG 32 KB) [file 12864_2014_6277_MOESM6_ESM.png]
